# Pharmacological Poly (ADP-Ribose) Polymerase Inhibitors Decrease *Mycobacterium tuberculosis* Survival in Human Macrophages

**DOI:** 10.3389/fimmu.2021.712021

**Published:** 2021-11-26

**Authors:** Cassandra L. R. van Doorn, Sanne A. M. Steenbergen, Kimberley V. Walburg, Tom H. M. Ottenhoff

**Affiliations:** Department of Infectious Diseases, Leiden University Medical Center, Leiden, Netherlands

**Keywords:** tuberculosis, host-directed therapy, poly (ADP-ribose) polymerase, rucaparib, human macrophages

## Abstract

Diabetes mellites (DM) is correlated with increased susceptibility to and disease progression of tuberculosis (TB), and strongly impairs effective global TB control measures. To better control the TB-DM co-epidemic, unravelling the bidirectional interactivity between DM-associated molecular processes and immune responses to *Mycobacterium tuberculosis* (*Mtb)* is urgently required. Since poly (ADP-ribose) polymerase (PARP) activation has been associated with DM and with *Mtb* infection in mouse models, we have investigated whether PARP inhibition by pharmacological compounds can interfere with host protection against *Mtb* in human macrophage subsets, the predominant target cell of *Mtb*. Pharmacological inhibition of PARP decreased intracellular *Mtb* and MDR-*Mtb* levels in human macrophages, identifying PARP as a potential target for host-directed therapy against *Mtb*. PARP inhibition was associated with modified chemokine secretion and upregulation of cell surface activation markers by human macrophages. Targeting LDH, a secondary target of the PARP inhibitor rucaparib, resulted in decreased intracellular *Mtb*, suggesting a metabolic role in rucaparib-induced control of *Mtb*. We conclude that pharmacological inhibition of PARP is a potential novel strategy in developing innovative host-directed therapies against intracellular bacterial infections.

## Introduction

Tuberculosis (TB) is an infectious disease caused by *Mycobacterium tuberculosis* (*Mtb*). Around 10 million new TB cases and 1.4 million deaths are reported annually and one quarter of the world’s population is latently infected with *Mtb* (1). Different risk factors have been identified for progression of latent TB infection (LTBI) to active disease, including age, malnutrition, coinfection with human immunodeficiency virus (HIV) or cytomegalovirus (CMV), and diabetes mellitus (DM) (1–3). In addition, recent studies have shown that DM is also associated with poorer clinical TB outcome following anti-bacterial treatment. Current estimates are that 15% of the global TB burden is now associated with DM (4, 5). Host-directed therapy (HDT) offers the potential for better treatment of Multi-Drug-Resistant (MDR)-TB, as well as shorten current standard (6-9 month) treatment regimens, thereby reducing toxicity, enhancing treatment compliance and as a result reducing emergence of *de novo* drug resistance. To better control the TB-DM co-epidemic, unravelling the bidirectional interactions between DM-dysregulated molecular processes and immune responses to *Mtb* is essential.

Macrophages (MFs) are known for their dual role in TB pathogenesis, as they provide a primary host niche for *Mtb* during infection while also being key host effector cells eliminating *Mtb*, and therefore represent key target cells in developing and evaluating novel therapeutic strategies for TB/DM, including innovative HDT. MFs are classically subdivided into pro-inflammatory macrophages (M1) and anti-inflammatory macrophages (M2), representing the polar ends of the macrophage spectrum. Both M1 and M2 have been reported in tuberculous granulomas, with M2 predominating in granulomas from patients with active TB (6).

Poly (ADP-ribose) polymerase (PARP) activation has been associated with cancer, diabetes and endothelial dysfunction in experimental mouse models and in humans (7–10). PARP-deficient mice and mice treated with PARP inhibitor PJ34 were resistant to streptozocin-induced diabetes, and pharmacological inhibition of PARP has been proposed for the treatment of DM (11–13). Interestingly, in addition, PARP inhibitors have recently been proposed as HDT compounds for reducing TB-induced inflammation (14), thereby providing a potential mechanistic link between the TB-DM co-morbidity at the molecular level. Several PARP inhibitors (PARPi) have been clinically approved for the treatment of cancer or are currently being evaluated in clinical trials for the treatment of advanced BRCA1/2 mutant ovarian and breast cancers, which can accelerate translation to HDTs against *Mtb*. PARP encompasses a family of enzymes involved in different cellular processes such as control of genomic stability, programmed cell death and DNA repair. When assisting in the repair of single-strand DNA nicks, PARP enzymes bind to single-stranded DNA breaks (SSB) or double-stranded DNA breaks (DSB) to generate PAR polymers on itself (auto-PARylation), histones and chromatin-associated proteins, which together leads to chromatin relaxation and recruitment of repair proteins. Inhibition of PARP results in accumulation of SSB due to delayed DNA repair. Given the dual role of PARP activation on diabetes and tuberculosis infection, we hypothesized that small molecule inhibitors that interfere with PARP activation at the cellular level may be able to redirect macrophage function in response to *Mtb* infection. Such chemical compounds could be valuable tools as part of immunomodulatory HDT regimens.

In the current study, we explore the potential of PARP inhibitors in the treatment of intracellular *Mtb* infections in human macrophages. We demonstrate that pharmacological inhibition of PARP decreases intracellular *Mtb* in human macrophages and modulates the immune response of *Mtb*-infected human macrophages. To our knowledge, the potential of targeting PARP for HDT in a human model of *Mtb*-infection has not been reported yet, and our work identifies PARPi as novel HDT compounds against *Mtb* and possibly other intracellular infectious diseases.

## Results

### Pharmacological Inhibition of PARP Reduces *Mtb* Survival in Human Macrophages in a Host-Directed Manner

**Figure 1:**
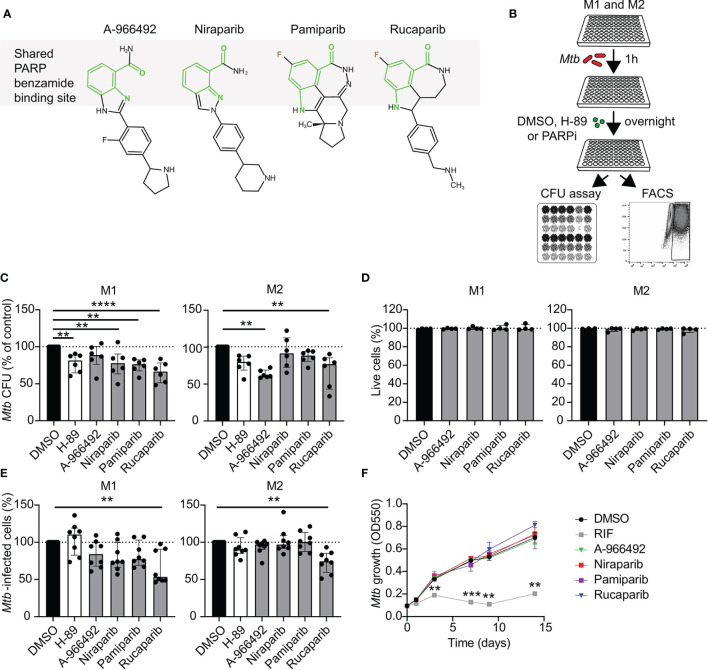
Identification of PARP as potential host target during *Mtb* infections in human macrophages. **(A)** Chemical structures of the PARPi used in this study. The benzamide moiety that is shared between all PARP inhibitor structures is highlighted in light green. **(B)** Schematic representation of the experimental setup used in C and E. **(C)** *Mtb* H37Rv-infected M1 and M2 were treated overnight with PARPi (10 μM), H-89 (10 μM) or an equal volume of vehicle control DMSO (0.1% v/v). CFU data represent the median ± interquartile range of six different donors. Dots represent the mean from triplicate wells of a single donor. CFUs are expressed as percentage of vehicle control (i.e. DMSO). Differences were significant by RM one-way ANOVA with Dunnett’s multiple test correction against DMSO. **(D)** Percentage of live M1 and M2 (i.e. PI-negative cells) after overnight treatment with PARPi (10 μM) or an equal volume of vehicle control DMSO (0.1% v/v). Data represent the median ± interquartile range from four different donors. Differences were tested by Friedman’s test with Dunn’s multiple test correction against DMSO. **(E)** Percentage of M1 and M2 infected with Venus-expressing *Mtb* H37Rv that were treated as in **(C)** Data represent the median ± interquartile range from eight different donors. Differences were significant by Friedman’s test with Dunn’s multiple test correction against DMSO. **(F)** Liquid *Mtb* H37Rv growth (in the absence of cells) was monitored for 14 days after addition of positive control RIF (20 µg/ml), PARPi (10 μM) or vehicle control DMSO (0.1% v/v). Data represent the means ± S.D. of triplicate wells from a representative experiment out of two independent experiments. Differences were significant by RM two-way ANOVA with Dunnett’s multiple test correction against DMSO. **p < 0.01.

To study whether PARP may play a role during *Mtb* infections in human macrophages, we first evaluated intracellular *Mtb* survival after treatment with four clinically relevant PARPi (**Figures 1A, B**). We selected 10 µM as a relatively high PARPi concentration, which has been shown to target PARP *in vitro* (15–17) and which allowed us to directly compare drug efficacy of PARPi with each other and with control kinase inhibitor H-89 (18, 19). Rucaparib significantly decreased intracellular *Mtb* CFUs in M1 and M2 more efficiently than H-89 (**Figure 1C**, n=6). Additionally, niraparib and pamiparib decreased intracellular *Mtb* in M1 and A-966492 decreased intracellular *Mtb* in M2, suggesting cell-specific effects of PARP inhibition on bacterial load for these compounds. Furthermore, we assessed the effect of PARPi on cytotoxicity using uninfected macrophages from four different donors, to ascertain that PARPi was not detrimental to healthy human macrophages. PARPi did not affect host-cell integrity and was not cytotoxic (**Figure 1D**). Next, we used a flow cytometry-based method (18) to evaluate whether the inhibition of *Mtb* outgrowth by PARPi was accompanied by a decrease in the percentage of cells harboring Venus-expressing *Mtb*. Rucaparib, but not other PARPi, significantly reduced the percentage of *Mtb*-infected cells both in M1 and M2 (**Figure 1E**, n=8), further corroborating the *Mtb*-inhibiting effect of rucaparib in human macrophages. To exclude that PARPi had direct microbicidal effects, liquid *Mtb* cultures were treated with PARPi in the absence of macrophages in two independent experiments (**Figure 1F**). None of the PARPi limited bacterial growth directly, indicating that PARPi restrict intracellular outgrowth of *Mtb* by modulating host pathways.

**Figure 2:**
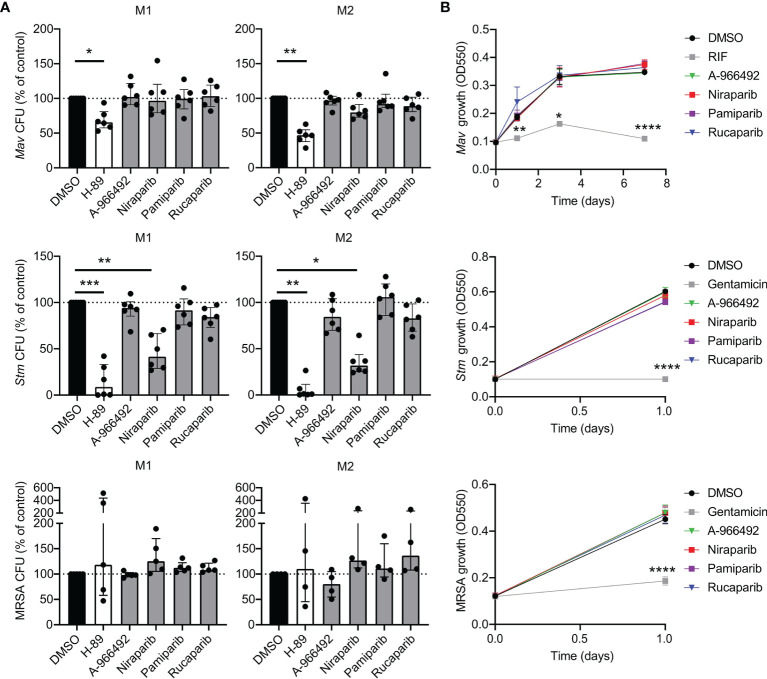
PARPi niraparib decreases intracellular *Stm*. **(A)** *Mav, Stm* and MRSA CFUs after overnight treatment of infected M1 and M2 with PARPi (10 μM), H-89 (10 μM) or an equal volume of vehicle control DMSO (0.1% v/v). Data represent the median ± interquartile range from at least four different donors. Dots represent the mean from triplicate wells of a single donor. CFUs are expressed as percentage of vehicle control (i.e. DMSO). Differences were significant by Friedman’s test with Dunn’s multiple test correction against DMSO. **(B)** Liquid *Mav, Stm* and MRSA growth (in the absence of cells) was monitored during treatment with RIF (20 µg/ml; positive control for *Mav*), gentamicin (50 µg/ml; positive control for *Stm* and MRSA), PARPi (10 μM) or vehicle control DMSO (0.1% v/v). Data represent the means ± S.D. of triplicate wells from a representative experiment out of two independent experiments. Differences were significant by RM two-way ANOVA with Dunnett’s multiple test correction against DMSO. *p < 0.05, **p < 0.01, ***, ****p < 0.0001.

To investigate whether PARPi-induced inhibition of intracellular bacterial growth was specific for *Mtb*, we evaluated whether pharmacological inhibition of PARP also restricted growth of other intracellular pathogenic bacteria, including *Mycobacterium avium* (*Mav*), *Salmonella enterica* serovar Typhimurium (*Stm*) and methicillin-resistant *Staphylococcus aureus* (MRSA). Interestingly, niraparib, but not other PARPi, significantly decreased *Stm* CFUs in M1 and M2 (**Figure 2A**, n=6) without exhibiting direct microbicidal effects in two independent experiments (**Figure 2B**). Rucaparib did not significantly diminish *Mav*, *Stm* or MRSA CFUs, suggesting that the effect of rucaparib is specific for *Mtb*. These results imply that both rucaparib and niraparib inhibit specific species of bacteria through modeling of host signaling pathways.

Taken together, these data suggest that PARP activity is involved in *Mtb* survival in human macrophages, and that although there is some difference in their efficacy against *Mtb*-infected M1 compared to *Mtb*-infected M2, pharmacological inhibition of PARP is a promising strategy to target intracellular *Mtb*. Moreover, the effects of different PARP inhibitors are highly specific to certain pathogen species.

### The Translational Potential of PARPi for Clinical Application

**Figure 3:**
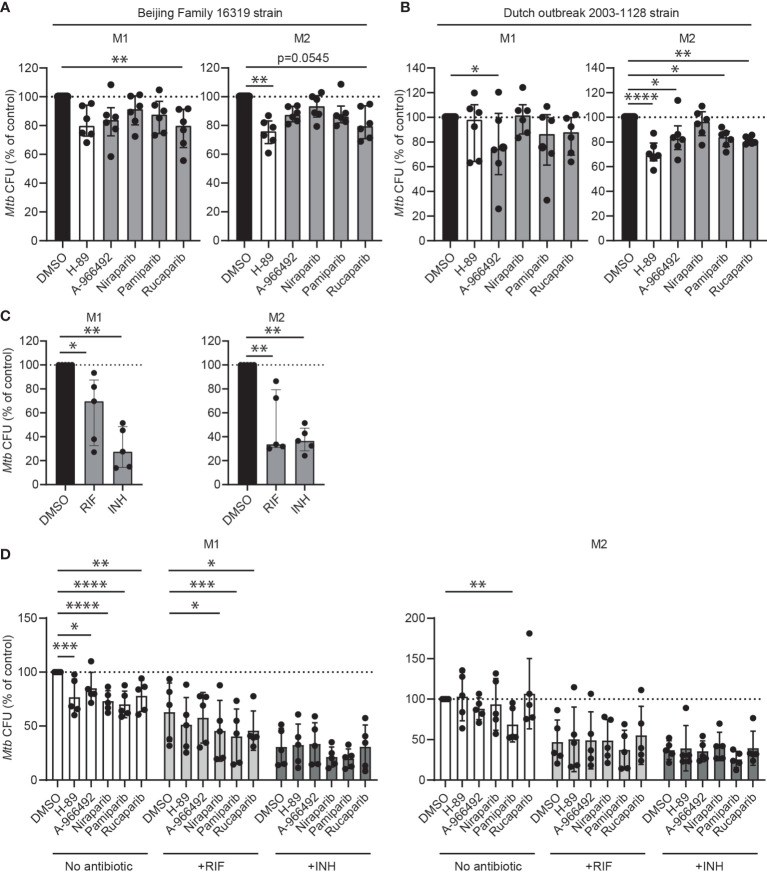
The clinical potential of PARPi for host-directed therapy against *Mtb*. **(A)** Multidrug-resistant (MDR) *Mtb* CFUs after overnight treatment with PARPi (10 μM), H-89 (10 μM) or an equal volume of vehicle control DMSO (0.1% v/v) in M1 and M2. Data represent the median ± interquartile range from six different donors. Dots represent the mean from triplicate wells of a single donor. CFUs are expressed as percentage of control (i.e. DMSO). Differences were significant by Friedman’s test with Dunn’s multiple comparison test against DMSO. **(B)** MDR-*Mtb* CFUs after overnight treatment with PARPi (10 μM), H-89 (10 μM) or an equal volume of vehicle control DMSO (0.1% v/v) in M1 and M2. Differences were significant by RM one-way ANOVA with Dunnett’s multiple comparison test against DMSO. **(C)** *Mtb* H37Rv CFUs after overnight treatment with RIF (0.05 μg/ml), INH (0.4 μg/ml) or an equal volume of vehicle control DMSO (0.1% v/v) in M1 and M2. Data represent the median ± interquartile range from five different donors. CFUs are expressed as percentage of control (i.e. DMSO). Differences were significant by RM one-way ANOVA with Dunnett’s multiple comparison test against DMSO. **(D)** *Mtb* H37Rv CFUs after overnight treatment with PARPi (10 μM), H-89 (10 μM) or an equal volume of vehicle control DMSO (0.1% v/v) in the presence or absence of a suboptimal dose of RIF (0.05 μg/ml) or INH (0.4 μg/ml) in M1 and M2. Data represent the median ± interquartile range from five different donors. CFUs are expressed as percentage of DMSO in the absence of antibiotics. Differences were significant by RM two-way ANOVA with Dunnett’s multiple comparison test against the DMSO controls in the presence or absence of antibiotics. *p < 0.05, **p < 0.01, ***p < 0.001, ****p < 0.001.

To evaluate the translational potential of PARPi for the development of HDT, we first tested whether PARPi displayed activity against macrophages infected with two different multi-drug resistant (MDR)-*Mtb* strains: an MDR-*Mtb* strain belonging to the Beijing genotype (strain 16319) and an MDR-*Mtb* Dutch outbreak strain (strain 2003-1128), both resistant to rifampicin (RIF) and isoniazid (INH). Although drug-susceptible and MDR *Mtb* strains are expected to respond similarly to HDT compounds, previous experiments by our laboratory suggested that in selective cases compound efficacy may differ between *Mtb* strains (20). Here, rucaparib significantly decreased intracellular MDR-*Mtb* and A-966492 showed activity against the MDR-*Mtb* Dutch outbreak strain (**Figures 3A, B**, n=6), highlighting the potential of PARPi to control intracellular outgrowth not only of drug-sensitive but also MDR-*Mtb* strains.

Next, the interaction of PARPi with first-line antibiotics was studied. Macrophages from five donors were infected with *Mtb* and treated with PARPi in the presence or absence of a non-toxic, suboptimal dose of RIF or INH (**Figure 3C**) (20, 21). As expected, PARPi significantly decreased *Mtb* CFUs in M1 (**Figure 3D**). In M2, however, several HDT compounds, including rucaparib, niraparib and positive control H-89 increased *Mtb* CFUs in two donors, illustrating the typically high variation in the response of donors to HDT compounds. Niraparib, pamiparib and rucaparib significantly enhanced the activity of INH against intracellular *Mtb* in M1. The coefficient of drug interaction (CID) of niraparib, pamiparib and rucaparib with RIF was close to 1 in M1, suggesting that these PARPi had an additive effect to the antibiotic treatment and not a synergistic effect (**Supplementary Table 1**). Furthermore, compared with other PARPi, pamiparib seemed to enhance the activity of RIF in M2 and of INH in M1 and M2, albeit not significant.

### PARPi Induce Immunomodulatory Effects in *Mtb*-Infected Human M1 and M2

**Figure 4:**
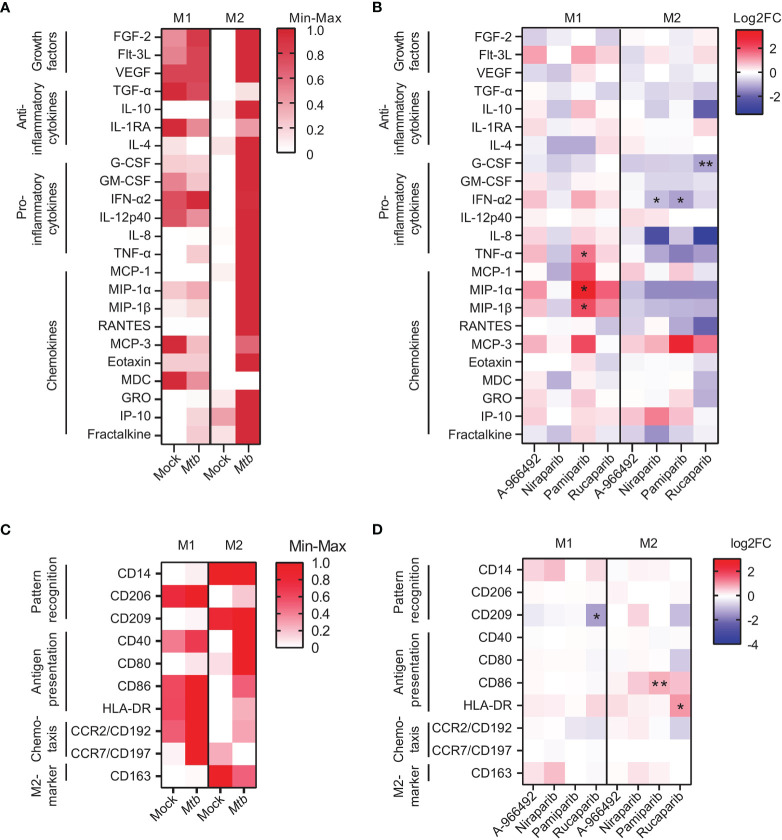
PARP inhibitors induce immunomodulatory effects in human macrophages. **(A)** Heatmap displaying median cytokine/chemokine levels in the supernatants of *Mtb* H37Rv- or mock-infected M1 and M2 obtained from eight donors. Shown are the relative cytokine/chemokine secretion levels (>10 pg/ml) on a white to red color scale (min=0; max=1). Differences were tested using a Wilcoxon matched-pairs signed rank test. **(B)** Heatmap displaying median log2 fold change (FC) cytokine/chemokine levels relative to vehicle control DMSO in the supernatants of *Mtb* H37Rv-infected M1 and M2 obtained from four donors. *Mtb* H37Rv-infected macrophages were exposed to PARPi (10 μM) or an equal volume of DMSO (0.1% v/v) overnight. Differences were significant by Friedman’s test with Dunn’s multiple comparison test against DMSO. **(C)** Heatmap displaying relative geometric mean fluorescence intensity (gMFI) of proteins on the surface of Venus-expressing *Mtb* H37Rv-infected M1 and M2 obtained from four donors. Shown are the relative (geometric mean fluorescence intensity) gMFI levels on a white to red color scale (min=0; max=1). Differences were tested using a Wilcoxon matched-pairs signed rank test. **(D)** Heatmap displaying log2 FC gMFI of proteins on the surface of Venus-expressing *Mtb* H37Rv-infected M1 and M2 obtained from four donors relative to vehicle control DMSO. Macrophages were exposed to PARPi (10 μM) or an equal volume of DMSO (0.1% v/v) overnight. Differences were significant by Friedman’s test with Dunn’s multiple comparison test against DMSO. *p < 0.05, **p < 0.01.

To investigate whether the effect of PARPi against *Mtb* correlated with macrophage activation during *Mtb* infection, cytokine and chemokine production was quantified in the supernatants of *Mtb*-infected macrophages that were treated with PARPi or vehicle control DMSO overnight. *Mtb*-infection induced cytokine and chemokine production in M1 and M2 and tended to induce tumor necrosis factor (TNF)-α, macrophage inflammatory protein (MIP)-1α,MIP-1β and IP-10 and fractalkine in M1 and M2 (**Figure 4A** and **Supplementary Table 2**, n=8). Exposure to PARPi altered the cytokine and chemokine response of M1 and M2 upon *Mtb* infection (**Figure 4B** and **Supplementary Table 2**, n=4): pamiparib and rucaparib both increased MIP-1α and MIP-1β secretion by M1 and significantly decreased IFN-α2 secretion by M2 compared to DMSO. Correlation plots between the cytokines and chemokines that were significantly modified by PARPi and *Mtb* CFUs did not identify a positive correlation between secretion profiles of macrophages and *Mtb* loads (**Supplemental Figure 1**). Collectively, these data suggest that pamiparib and rucaparib can modify the cytokine and chemokine response to *Mtb* infection.

Next, we investigated the expression levels of activation markers on the surface of *Mtb*-infected M1 and M2 that were treated with PARPi overnight. *Mtb*-infection tended to increase macrophage activation markers in M1 and M2 (**Figure 4C**, n=4). Surprisingly, rucaparib, but not other PARPi, tended to decrease expression of CD192 and CD209 and expression of human leucocyte antigen (HLA)-DR on the surface of *Mtb*-infected M1 and M2 (**Figure 4D** and **Supplemental Figure 2**, n=4). Furthermore, the expression of costimulatory molecule CD86 on the surface of M2 tended to be increased by rucaparib, and was significantly increased by pamiparib. These data suggest that rucaparib increases macrophage activation and possibly antigen presentation.

Given that several HDT compounds with demonstrated efficacy against intracellular *Mtb* in macrophages exert their activity by activating autophagy (22–25), we next assessed whether treatment with PARPi induced autophagy and lysosomal maturation. Accumulation of autophagic and lysosomal vesicles was quantified by confocal imaging on *Mtb*-infected and PARPi-treated macrophages from four donors that were stained with CYTO-ID or Lysotracker, respectively. Formation of autophagic or lysosomal vesicles could not be detected after 4h treatment with PARPi compared to DMSO (**Supplemental Figures 3A, B**). To examine the effect of PARPi on the autophagic flux, macrophages from three donors were treated with PARPi in the presence or absence of bafilomycin A1 (Baf) which is an autophagy inhibitor that prevents fusion between autophagosomes and lysosomes. As expected, treatment with Baf induced microtubule-associated protein light chain 3 (LC3)-II accumulation in M1 and M2 (**Supplemental Figure 3C**). However, PARPi did not increase LC3-II protein levels, regardless of the presence of Baf. Also, PARPi did not increase LAMP1 protein levels, suggesting that PARPi do not act *via* induction of autophagic and/or lysosomal pathways to control intracellular *Mtb* in human macrophages.

Collectively, our data demonstrate that PARPi modulate the immune response of *Mtb*-infected human macrophages *via* cytokine/chemokine expression and surface markers, which play a role in antigen presentation and chemotaxis. Our data also suggest that a role of induction of autophagy and phagosome maturation in PARP-induced *Mtb* control is not likely mechanistically involved in the mode of action.

### Lactate Dehydrogenase, a Secondary Target of Rucaparib, Induces *Mtb* Control in Human Macrophages

**Figure 5:**
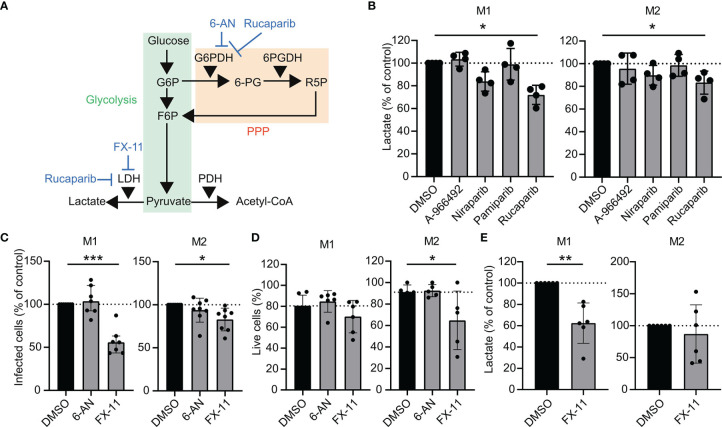
Increased *Mtb* control by pharmacological inhibition of lactate dehydrogenase in human macrophages. **(A)** Schematic representation of metabolic pathway modulation by rucaparib, 6-AN and FX-11. Glycolysis is depicted in green; the pentose phosphate pathway (PPP) is depicted in orange. **(B)** Determination of lactate production in the supernatants of *Mtb* H37Rv-infected M1 and M2 that were treated with PARPi (10 µM) or an equal volume of vehicle control DMSO (0.1% v/v) overnight. Data represent the median ± interquartile range from four different donors. Dots represent the mean from duplicate wells of a single donor. Differences were significant by RM one-way ANOVA with Dunnett’s multiple comparison test against DMSO. **(C)** Percentage of M1 and M2 infected with Venus-expressing *Mtb* H37Rv that were treated with G6PD-inhibitor 6-aminonicotinamide (6-AN, 100 µM) or LDH-inhibitor FX-11 (100 µM) or an equal volume of vehicle control DMSO (0.1% v/v) overnight. Data represent the median ± interquartile range from at least seven different donors. Differences were significant by RM one-way ANOVA with Dunnett’s multiple comparison test against DMSO. **(D)** Percentage of live M1 and M2 (i.e. PI-negative cells) after overnight treatment with 6-AN (100 μM) or FX-11 (100 μM) or an equal volume of vehicle control DMSO (0.1% v/v). Data represent the median ± interquartile range from at least five different donors. Differences were significant by RM two-way ANOVA with Dunnett’s multiple test correction against DMSO. **(E)** Determination of lactate production in the supernatants of *Mtb* H37Rv-infected M1 and M2 that were treated with FX-11 (100 µM) or an equal volume of vehicle control DMSO (0.1% v/v) overnight. Data represent the median ± interquartile range from six different donors. Differences were significant by RM one-way ANOVA with Dunnett’s multiple comparison test against DMSO. *p < 0.05, **p < 0.01, ***p < 0.001.

Hexose-6-phosphate dehydrogenase (H6PD) and lactate dehydrogenase (LDH) were recently identified as additional target molecules of rucaparib (26). We therefore hypothesized that these metabolic targets might play a role in the more profound inhibitory effect of rucaparib on *Mtb* survival compared to other PARPi (**Figures 1C, E**, **3A**, **5A**). Lactate levels were significantly decreased in the supernatants of *Mtb*-infected M1 and M2 that had been treated with rucaparib compared to DMSO, suggesting that rucaparib indeed impaired LDH activity (**Figure 5B**, n=4). Interestingly, none of the other selected PARPi significantly affected lactate levels, compatible with the fact that none of these have been reported to modulate LDH activity. To study whether inhibition of H6PD or LDH could have contributed to the effect of rucaparib on *Mtb* control, we treated *Mtb*-infected macrophages with the specific LDH-inhibitor FX-11 or the G6PD-inhibitor 6-aminonicotinamide (6-AN) and determined intracellular *Mtb* levels in a flow cytometry-based assay, which allows a more rapid quantification of intracellular *Mtb* levels compared to classical CFU assays and generally showed a greater effect window of rucaparib compared to CFU assay (**Figure 1C** versus **1E**). Interestingly, FX-11 significantly decreased the percentage of *Mtb*-infected M1 (n=7) and M2 (n=8), whereas no effect of 6-AN on intracellular *Mtb* could be detected (**Figure 5C**). FX-11 did not affect the percentage of live M1 cells (n=6), but did decrease the percentage of live cells in a subset of M2 cultures (n=5). Together, these data suggest that inhibition of LDH, but not G6PD, likely contributed to the *Mtb*-decreasing effect of rucaparib. Corroborating this hypothesis, FX-11 treatment significantly decreased lactate levels in the supernatant of *Mtb*-infected M1, corresponding with decreased LDH activity (**Figure 5E**, n=6). FX-11 treatment did not result in decreased lactate levels in M2, which is likely a result of cellular toxicity in M2 (**Figures 5D, E**). Collectively, these data suggest that LDH could be involved in rucaparib-induced inhibition of *Mtb* and identifies LDH as potential host target during *Mtb* infections.

## Discussion

In search of new potential treatments against TB, we have studied a series of PARPi, which are being considered for treatment of type 2 diabetes, for their ability to improve intracellular growth control by infected human M1 and M2 as novel candidate HDT therapeutics. Here, we showed that clinical compounds targeting PARP were able to decrease the intracellular survival of *Mtb*, suggesting that pharmacological inhibition of PARP can be a novel tool for TB treatment. PARPi had no direct antimycobacterial activity, suggesting that PARPi act in a HDT like fashion. In line with this, macrophage treatment with PARPi was associated with the modulation of macrophage function at the cytokine level and surface marker level. Lactate dehydrogenase (LDH) was identified as an additional target molecule for rucaparib’s activity against intracellular *Mtb* which might explain the more profound inhibitory effect of rucaparib on *Mtb* survival compared to other PARPi. To our knowledge, the potential of PARPi as HDTs against *Mtb* has not been demonstrated before.

PARPs are host proteins involved in cellular processes acting *via* PARylation of their targets. It has recently been shown that PARP1 activation is triggered in mouse lungs upon *Mtb* infection (27). Importantly, genetic PARP1 depletion resulted in decreased lung bacterial burden in female mice. Here, we studied the role of PARP inhibition in a human and clinically relevant context. All clinical PARPi used in this study are known PARP1 and PARP2 inhibitors and have the ability to inhibit multiple proteins of the PARP family (28–31). Our data suggest that the efficacy of PARPi on bacterial clearance is specific for *Mtb* and does not translate to other intracellular bacteria, including the closely related species *Mav*. While not investigated here, this suggests that PARP is not a key target for MRSA*, Stm* and *Mav*, although the exact role of PARP during other bacterial infections remains to be resolved.

Several PARP inhibitors, such as olaparib and talazoparib, can be metabolized by the hepatic enzyme CYP3A4, which is induced by the first-line TB antibiotic RIF (32–34). Rucaparib, however, is less sensitive to metabolization by CYP3A4 and the sensitivity of other PARPi remains to be determined. In our *in vitro* infection model, pamiparib had a significant additive effect to RIF in M1 and seemed to have an additive effect to RIF in M2 and to INH in both M1 and M2. This possible additive effect of pamiparib to RIF and INH in M2 was unexpected, since pamiparib did not show activity against *Mtb* in the absence of antibiotics in these cells. Although we observed differences in the cellular response to pamiparib in M1 versus M2 based on cytokine/chemokine secretion, it remains currently undefined which exact mechanisms were responsible for the difference in the efficacy of pamiparib against *Mtb* in M1 versus M2. This requires further investigation.

In addition to their role in DNA repair, mammalian PARPs display properties that are associated with and are likely to impact host-pathogen interactions. Firstly, PARPs are induced by the production of interferon-stimulated genes (ISGs), implicating a role during virus infection (35–38). The impact of PARP activity on virus replication remains controversial, as both promotion and reduction of viruses by PARP have both been described, which is likely dependent on the virus (as reviewed by (39). Moreover, an elevated type I IFN response correlates with progression to active TB in humans (40–42) and has been implicated in susceptibility to *Mtb* in mouse models (43, 44). Secondly, PARPs are involved in pro-inflammatory gene expression. Macrophages from PARP-deficient mice were unable to induce nuclear factor kappa B (NF-κB)-mediated activation in response to lipopolysaccharide (LPS), limiting inducible nitric oxide synthase (iNOS), tumor necrosis factor alpha (TNF-α) and interferon gamma (IFN-γ) levels (45, 46). Here, we show that the inflammatory response of *Mtb*-infected macrophages is modified by pharmacological inhibition of PARP. At the cytokine level, pamiparib and rucaparib induced expression of chemokines MIP-1α and MIP-1β in M1. We furthermore show that the higher MIP-1α and MIP-1β levels did not correlate directly with increased inhibition of intracellular *Mtb*, compatible with their primary role in chemotactic cellular recruitment. Decreased MIP-1α expression is associated with diabetes in *Mtb*-infected human monocyte-derived macrophages and in *Mtb*-infected diabetic mice (47–49). This suggests that macrophages obtained from diabetic patients or mice may be impaired in the recruitment of immune cells to *Mtb* sites of infection. Additionally, alveolar macrophages of diabetic mice feature decreased TNF-α secretion (47). Mice that have impaired TNF-α secretion and mice and humans treated with anti-TNF-α antibodies are more susceptible to *Mtb* infection, highlighting a direct protective effect of TNF-α against *Mtb* (50–52). Here, TNF-α was significantly induced by pamiparib treatment and it tended to be induced by A-966492 and rucaparib in M1. However, like for MIP-1α, higher TNF-α levels did not correlate directly with decreased intracellular *Mtb* levels. Together, these data demonstrate that PARPi treatment alters the cytokine/chemokine response of human macrophages during infection with *Mtb.* These effects are not directly correlated with intracellular *Mtb* control by macrophages, but these cytokines/chemokines could exert effects *in vivo via* other cells such as recruitment of immune cells. The potential of PARPi to improve recruitment of immune cells to sites of infection remains to be elucidated and could play an important role in the *in vivo* activity of PARPi against *Mtb*.

When evaluating the activation status of *Mtb*-infected macrophages in response to PARPi treatment, rucaparib appeared to increase the expression of HLA-DR and CD86, in contrast to other PARPi. As previously published, monocyte-derived macrophages (MDMs) obtained from type 2 diabetes (T2D) patients displayed lower levels of HLA-DR and CD86 compared to MDMs obtained from healthy individuals upon *n vitro Mtb* infection (48). Collectively, these observations support the hypothesis that rucaparib is able to reverse diabetes-induced functional effects of macrophages and suggest that rucaparib could increase antigen presentation by human macrophages to specific T-cells.

Several studies have shown that diabetes was associated with a decrease in *Mtb* phagocytosis capacity in human and murine macrophages (47, 48, 53), possibly resulting from highly glycosylated proteins that impair recognition of bacterial components. Phagocytosis as mechanism of action of PARPi can be excluded in our current study, since PARPi were added well after phagocytosis, and further cell-to-cell exchange of *Mtb* bacteria was prevented by supplementing the culture medium with low dose gentamicin.

Multiple PARPi, including niraparib, rucaparib and talazoparib have recently been described to induce autophagy in cancer cell lines, as detected by increased LC3-I to LC3-II conversion on western blot analysis (54, 55). In our current study, induction of autophagy could not be detected, however, perhaps because non replicating primary cells (M1, M2) were used instead of cell lines.

Proteome and metabolome profiling have recently revealed additional targets for PARPi which could contribute to *Mtb* control in our human macrophage infection model. *In vitro* kinase profiling showed that olaparib and rucaparib have a relatively similar affinity for members of the PARP family, but differ substantially in their affinity for protein kinases (29, 56). Our observation that rucaparib performed significantly better against *Mtb* than other PARPi suggests that unique kinases targeted by rucaparib might contribute to its effect against *Mtb.* The reported unique additional targets of rucaparib include cyclin dependent kinase 1 and 16 (CDK1, CDK16), pim-3 oncogene (PIM3), hexose-6-phosphate dehydrogenase (H6PD) and lactate dehydrogenase (LDH) (26, 28, 56, 57). Here we show that rucaparib decreased lactate production in *Mtb*-infected M1 and M2. Moreover, the specific LDH inhibitor FX-11 had a similar effect as rucaparib on intracellular *Mtb* load. This suggests that LDH inhibition could be the major mechanism of action of rucaparib, and as such could be a promising target pathway in developing HDT against *Mtb*. In agreement with our findings, oral administration of FX-11 was recently described to decrease intracellular *Mtb* levels in mouse BMDM in a host-directed manner (58). Based on our data, we cannot exclude additional effects of rucaparib on further (secondary) targets during *Mtb* infection in human macrophages. Similarly, studying the potentially additive effects of the already described unique additional targets of niraparib are interesting in the development of novel HDT against *Mav* and *Stm*. Deoxynucleoside kinase (DCK) is a reported additional target of niraparib, and analysis of a published dataset of infected M2 showed that DCK is significantly upregulated 4h post *Stm*, but not *Mtb* infection (56, 59). Taken together, these data suggest that DCK is an interesting candidate for HDT against *Stm*.

There are several limitations of the current study. First, the current study has the limitation of small sample size. Although a small sample is common among studies with (primary) cells due to the laborious nature of these experiments, this could have resulted in insufficient statistical power between groups. Furthermore, we applied a single-dose administration of HDT compounds with a relatively short effect time (i.e. after overnight infection), which is an experimental setup that we generally use for the identification of novel HDT compounds (18, 20, 60). Although applying other compound concentrations, repeated doses or a longer treatment exposure could have resulted in a bigger effect size, the host targets (PARP and LDH) that we identified in our *in vitro* human macrophage model were recently published as host targets using *in vivo* mouse models as discussed above (27, 58). This agreeing set of independent findings underscores the power and validity of our approach, which aims to decipher novel pathways involved in host defense against intracellular bacterial infections in humans, and develop corresponding novel HDT.

In summary, we identified PARP as a host target for HDT during *Mtb* infections, and confirmed that PARP inhibition is a promising avenue in the development of HDT against *Mtb*. Furthermore, our data show that also off-target kinase pharmacology of PARP inhibitors may expand the current clinical scope of PARP inhibitors and that these molecules deserve serious consideration in the development of repurposed HDT against intracellular bacterial infections.

## Materials and Methods

### Reagents and Antibodies

Dimethyl sulfoxide (DMSO), staurosporine solution from Streptomyces sp. (STSP), H-89 dihydrochloride hydrate (H-89), bafilomycin A1 (Baf), 6-aminonicotinamide (6-AN), lactate dehydrogenase A inhibitor (FX-11), rifampicin (RIF), gentamicin, tetracycline hydrochloride, ethylenediaminetetraacetic acid (EDTA) and Hoechst 33342 were obtained from Sigma-Aldrich (Zwijndrecht, The Netherlands). PARP inhibitors (A-966492, niraparib, pamiparib and rucaparib) and isoniazid (INH) were purchased from SelleckChem.

PE anti-human CD209 (1:50, clone 9E9A8), PerCP/Cy5.5 anti-human CD192 (CCR2, 1:20, clone K036C2), PE/Cy7 anti-human CD40 (1:100, clone 5C3), Alexa Fluor^®^ 647 anti-human CD163 (1:50, clone CHI/61), APC/Cy7 anti-human CD206 (1:50, clone 15-2) and BV785 anti-human CD14 (1:200, clone M5E2) were purchased from Biolegend (London, United Kingdom). APC-R700 anti-human CD80 (1:200, clone L307.4), V500 anti-human HLA-DR (1:100, clone G46-6) and BV711 anti-human CD86 (1:100, clone 2331) were purchased from BD Bioscience (Vianen, The Netherlands). BV605 anti-human CD197 (CCR7, 1:200, clone G043H7) was purchased from Sony Biotechnology (Weybridge, United Kingdom). Mouse monoclonal anti-human beta actin (1:2000, clone mAbcam 8226) and rabbit polyclonal anti-human LAMP1 (1:500) were purchased from Abcam (Amsterdam, The Netherlands). Rabbit polyclonal anti-human LC3-I/II (1:250) was purchased from Cell Signaling Technology (Leiden, The Netherlands). Secondary stabilized peroxidase conjugated antibodies goat anti-rabbit IgG (H+L) (1:10,000) and goat anti-mouse IgG (H+L) (1:10,000) were purchased from Thermo Fisher Scientific (Breda, The Netherlands).

### Cell Culture

Human peripheral blood mononuclear cells (PBMCs) were isolated from buffy coats of healthy anonymous donors (Dutch, adults) after written informed consent (Sanquin Blood Bank, Amsterdam, The Netherlands) by density gradient centrifugation over Ficoll/amidotrizoaat as described earlier (61). The use of buffy coats for research purposes was approved by the Institutional Review Board of the Leiden University Medical Center, The Netherlands. Magnetic cell sorting using anti-CD14-coated microbeads (Miltenyi Biotec, Auburn, CA) was used to isolate CD14+ monocytes. CD14+ cells were cultured for six days at 37°C/5% CO_2_ in Gibco Roswell Park Memorial Institute (RPMI) 1640 medium (Thermo Fisher Scientific) supplemented with 10% fetal bovine serum (FBS), 2 mM L-Alanyl-L-Glutamine (PAA, Linz, Austria), 100 units/ml penicillin (Thermo Fisher Scientific), 100 µg/ml streptomycin (Thermo Fisher Scientific) and either 5 ng/ml granulocyte-macrophage colony-stimulating factor (GM-CSF, Thermo Fisher Scientific) to promote M1 differentiation or 50 ng/ml macrophage colony-stimulating factor (M-CSF, R&D Systems, Abingdon, United Kingdom) to promote M2 differentiation. Cytokines were added again at day 3 of differentiation in equal concentrations. The M1 and M2 macrophage phenotypes were subsequently validated by flow cytometry based surface marker expression (M1: CD14^low^, CD163^low^, CD11b^high^; M2: CD14^high^, CD163^high^, CD11b^low^) and by quantifying IL-10 and IL-12p40 production by Enzyme-Linked Immuno Sorbent Assay (ELISA) following stimulation of cells in the presence or absence of 100 ng/ml lipopolysaccharide (LPS) for 24h (*In vivo*Gen, San Diego, United States) as described before (60).

### Bacterial Culture

Mycobacterial strains were cultured at 37°C in Difco™ Middlebrook 7H9 Broth (BD Bioscience) containing 10% acid-albumin-dextrose-catalase (ADC, BD Bioscience), 0.5% Tween-80 (Sigma-Aldrich), 2% Glycerol (Sigma-Aldrich) and 50 µg/ml hygromycin B (Thermo Fisher Scientific) when appropriate. The following bacterial strains were used: *Mtb* H37Rv, Venus-expressing *Mtb* H37Rv (strain mc^2^8120), DsRed-expressing *Mtb* H37Rv (18), MDR-*Mtb* Beijing family strain 16319 (62), MDR-*Mtb* Dutch outbreak strain 2003-1128 (62), *Mycobacterium avium* subsp. *avium* Chester strain 101 (ATCC, Wesel, Germany), DsRed-expressing *Salmonella enterica* serovar Typhimurium (*Stm*) strain SL1344 (18) and GFP-expressing methicillin-resistant *Staphylococcus aureus* (MRSA) strain USA300 JE2.

### *In Vitro* Infection and Compound Treatment

Adherent cells were harvested by trypsinization and gentle scraping in FBS and seeded in RPMI 1640 medium supplemented with 10% FBS and 2 mM L-Alanyl-L-Glutamine on Costar 96-well flat bottom culture plates (30,000 cells/well) or on Costar 24-well flat bottom culture plates (300,000 cells/well) (Corning, Amsterdam, The Netherlands) and incubated overnight at 37°C/5% CO_2_. Mycobacterial cultures were diluted to an early log-phase corresponding with an OD600 of 0.25 one day prior to infection in 7H9 broth containing 10% ADC, 0.5% Tween-80 and 2% Glycerol. DsRed-expressing *Stm* was recovered from frozen stock and cultured in Difco™ Luria-Bertani (LB) broth (BD Bioscience) containing 100 µg/ml ampicillin (Sigma-Aldrich) overnight at 37°C. Bacterial suspension was diluted 1:33 in LB broth and grown for 3-4h to reach a log-phase with an OD600 between 0.4-0.6. GFP-expressing MRSA was recovered from frozen stock and cultured in Tryptic Soy (TS) broth (BD Bioscience) containing 5 µg/ml tetracycline hydrochlorine overnight at 37°C. Bacterial suspension was diluted 1:33 in TS broth and grown for 2-3h to reach a log-phase with an OD600 between 0.4-0.6. MRSA was harvested by centrifugation and resuspended in cold PBS supplemented with 5 mM EDTA. The indicated bacteria were diluted in RPMI 1640 medium (10% FBS and 2 mM L-Alanyl-L-Glutamine) to reach a multiplicity of infection (MOI) of 10. Accuracy of the MOI was validated by plating a serial dilution of the mycobacterial inoculum on Difco™ Middlebrook 7H10 agar (BD Bioscience) plates containing 10% oleic acid-albumin-dextrose-catalase (OADC, BD Bioscience) and 5% glycerol, by plating *Stm* on Difco™ LB agar plates (BD Bioscience) or by plating MRSA on TS agar (BD Bioscience). For mock infections, 7H9 broth was diluted in RPMI 1640 medium (10% FBS and 2 mM L-Alanyl-L-Glutamine) in equal concentrations (v/v) as the infection inoculum. Cells in flat-bottom 96-well plates containing 100 µl RPMI 1640 medium (10% FBS and 2 mM L-Alanyl-L-Glutamine) per well were inoculated with 100 µl bacterial suspension or mock solution. Cells in flat-bottom 24-well plates containing 500 µl RPMI 1640 medium (10% FBS and 2 mM L-Alanyl-L-Glutamine) per well were inoculated with 500 µl bacterial suspension or mock solution. Plates were centrifuged for 3 min at 800 rpm to increase bacterial uptake and incubated for 1h for *Mtb, Mav* or MRSA infection or for 20 minutes for *Stm* infection at 37°C/5% CO_2_. Extracellular bacteria were removed by washing with fresh RPMI 1640 medium supplemented with 10% FBS, 2 mM L-Alanyl-L-Glutamine and 30 µg/ml gentamicin sulphate for 10 min. Cells were incubated at 37°C/5% CO_2_ in RPMI 1640 medium supplemented with 10% FBS and 2 mM L-Alanyl-L-Glutamine and 5 µg/ml gentamicin sulphate in the presence of H-89 (10 µM), A-966492 (10 µM), niraparib (10 µM), pamiparib (10 µM), rucaparib (10 µM), 6-AN (100 µM), FX-11 (100 µM), Baf (10 nM), INH (0.4 μg/ml), RIF (0.05 μg/ml) or an equal amount of vehicle control DMSO (0.1% v/v) until readout. To calculate the coefficient of drug interaction (CID) the following formula was used: CID = AB/(AxB), where A indicates the ratio between antibiotic to the control group (DMSO, without antibiotics), B indicates the ratio between HDT compound to the control group and AB indicates the ratio between the treatments combined to the control group (63). A CID of 1 indicates an additive effect, <1 a synergistic effect and >1 an antagonistic effect.

### CFU Assay

Cells in 96-well flat bottom plates (30,000 cells/well) were washed with PBS and lysed in 0.05% sodium dodecyl sulfate (SDS) solution (Thermo Fisher Scientific). Serially diluted cell lysates were plated on 7H10 Agar containing 10% OADC and 5% Glycerol (*Mtb* and *Mav*), LB agar (*Stm*) or TS agar (MRSA) and incubated at 37°C. CFUs were determined from triplicate wells.

### Cellular Toxicity Assay

The number and percentage of dead cells based on plasma membrane integrity of the adherent cell population was quantified by analysis of microscopy images. Cells in 96-well flat bottom plates (30,000 cells/well) were stained with 2 µg/ml propidium iodide (Sigma-Aldrich) and 2 µg/ml Hoechst 33342 (H3570, Sigma-Aldrich) in 40 µl/well phenol red-free RPMI (Sigma-Aldrich) supplemented with 10% FBS and 2 mM L-Alanyl-L-Glutamine and incubated for 5 min at room temperature (RT). Cells were imaged using a Leica AF6000 LC fluorescence microscope (Leica Microsystems, Wetzlar, Germany) combined with a 10x dry objective. Total and dead cell numbers were quantified by respectively counting the nuclei and the number of propidium iodide-positive cells using ImageJ software (64). STSP (2.5 µM) was included as a positive control for cell death (**Supplemental Figure 4**).

### Extracellular Bacterial Growth Assay

Compounds were diluted in Difco™ Middlebrook 7H9 Broth (*Mtb*, *Mav*), Difco™ LB Broth (*Stm*) or TS Broth (MRSA) and were added to log-phase bacterial cultures in flat bottom 96-well plates (OD_600_ of 0.1). RIF (20 µg/ml) was added as positive control for reduction of *Mtb* and *Mav* growth and gentamicin (50 µg/ml) was added as positive control for reduction of *Stm* and *MRSA* growth. Bacterial plates were incubated at 37°C. Absorbance was measured directly after plating and at indicated times at a 550 nm wavelength on a Mithras LB 940 plate reader (Berthold Technologies, Bad Wildbad, Germany).

### Western Immunoblot Analysis

Cells (300,000 cells/well in 24-wells plates) were lysed with 100 µl/well EBSB buffer (10% v/v glycerol, 3% SDS, 100 mM Tris-HCl, pH 6.8) supplemented with one tablet of cOmplete™ EDTA-free protease inhibitor cocktail (Sigma-Aldrich) and one tablet of phosphatase inhibitor cocktail (PhosSTOP EASYpack, Sigma-Aldrich) per 10 ml. Cell lysates were boiled for 10 minutes at 95°C and stored at -20°C until use. Total protein concentrations were determined using a Pierce™ BCA protein assay kit (Thermo Fisher Scientific) according to manufacturer’ instructions and protein concentrations were equalized and diluted in Laemmli sample buffer (Biorad) containing β-mercaptoethanol (Sigma-Aldrich). Samples were loaded on a 15-well 4–20% Mini-PROTEAN^®^ TGX™ Precast Protein Gel (Bio-Rad Laboratories, Veenendaal, the Netherlands) and Amersham ECL Rainbow Molecular Weight Marker was added as reference (Sigma-Aldrich). Proteins were transferred to methanol-activated Immun-Blot PVDF membranes (Biorad) in Tris-glycine buffer (25 mM Tris, 192 mM glycine and 20% methanol). Membranes were blocked for 1h in polysorbate 20 tris-buffered saline (TTBS) supplemented with 5% w/v non-fat dry milk and incubated with the indicated antibodies in 5% non-fat dry milk/TTBS overnight at 4°C. Membranes were washed thrice for 15 min in TTBS and stained with secondary antibodies in 5% non-fat dry milk/TTBS for 2h at RT. Membranes were washed for 30 min with TTBS prior to revelation using enhanced chemiluminescence (ECL)™ Prime Western Blotting System reagent (GE Healthcare, Hoevelaken, The Netherlands). Imaging was performed on ChemiDoc XRS+ (Bio-Rad) or on an iBright Imaging System (Invitrogen, Breda, The Netherlands). Protein bands were quantified using ImageJ/Fiji software (64) and normalized to actin.

### Cytokine and Chemokine Secretion by Multiplex Beads Assay

Human cytokine/chemokine levels were determined using the MilliPlex Human Cytokine/Chemokine magnetic bead premixed 41-plex kit (Millipore Billerica, MA, USA) as described before (60). Culture supernatants were collected and sterilized by using a 96-well filter plate containing a 0.2 µm PVDF membrane (Corning) following centrifugation. The following analytes were measured on a Bio-Plex 100 with Bio-Plex ManagerTM software v6.1 (Biorad): sCD40L, EGF, FGF-2, Flt3 ligand, Fractalkine (CX3CL1), G-CSF, GM-CSF, GRO (CXCL1), IFN-γ, IFN-α2, IL-1α, IL-1β, IL-1RA, IL-2, IL-3, IL-4, IL-5, IL-6, IL-7, IL-8 (CXCL8), IL-9, IL-10, IL-12p40, IL-12p70, IL-13, IL-15, IL-17a, IP-10 (CXCL10), MCP-1 (CCL2), MCP-3 (CCL7), MDC (CCL22), MIP-1α (CCL3), MIP-1β (CCL4), PDGF-AB/BB, RANTES (CCL5), TGF-α, TNF-α, TNF-β, VEGF, Eotaxin (CCL11) and PDGF-AA.

### Flow Cytometry

Adherent and floating cells (300,000 cells/well in 24-wells plates) were washed in PBS/0.1% BSA (Merck, Darmstadt, Germany) and Fc receptors were blocked with 5% human serum (HS) in PBS for 10 min at RT. Cells were washed in PBS/0.1% BSA and stained with antibodies diluted in PBS/0.1% BSA for 30 min at 4°C. Cells were washed in PBS/0.1% BSA and fixed with 1% paraformaldehyde for 1h at 4°C. Fluorescence minus one (FMO) control samples were included to define background fluorescence for each stain. Fluorescence staining was measured using a FACSLyric™ flow cytometer with FACSDiva software (BD Bioscience). Geometric mean fluorescence intensity (gMFIs) was recorded for each sample. Data were analyzed using FlowJo v10 software.

### Lactate Assay

Cell culture supernatants were collected and sterilized by using a 96-well filter plate containing a 0.2 µm PVDF membrane (Corning) following centrifugation. Undiluted sodium l-lactate (Sigma-aldrich) standard (0-8 mM, 5 µl) or sample (5 µl) was added to a flat-bottom 96-well plate (Greiner Bio-One, Alphen a/d Rijn, The Netherlands). 200 µl of reaction mix (0.74 mM NAD, Roche Applied Science, Woerden, The Netherlands; 0.4 mM glycine, Sigma-Aldrich; 0.4 M hydrazine hydrate, Sigma-Aldrich) was added to allow conversion of lactate to pyruvate by lactate dehydrogenase (LDH): Lactate + NAD^+^ <–> Pyruvate + NADH + H^+^. Hydrazine hydrate was added to avoid conversion of the newly formed pyruvate back to lactate. Baseline NADH levels were measured using the SpectraMax i3x plate reader at OD340 before the addition of 2 µl of three times diluted LDH from rabbit muscle (Roche Applied Science) to each well to initiate the lactate to pyruvate conversion. Following the addition of LDH, plates were incubated on a shaker at RT for 90 mins and the OD340 was measured again using a SpectraMax i3x plate reader.

### Immunostaining and Confocal Microscopy

For confocal microscopy, cells were cultured on pre-washed black glass bottom poly-d-lysine coated 96-well plates (no. 1.5, MatTek Corporation, Ashland, MA, USA) at a density of 30,000 cells/well in RPMI 1640 medium supplemented with 10% FBS and 2 mM L-Alanyl-L-Glutamine and incubated overnight at 37°C/5% CO_2_. Cells were infected with DsRed-expressing *Mtb* H37Rv as described above and treated with compound (10 µM) or an equal volume of DMSO (0.1% v/v) for 4h. Culture medium was replaced with a solution of 75 nM LysoTracker^®^ Deep Red (Thermo Fisher Scientific) and 1x CYTO-ID^®^ Green 2.0 (1:500, Enzo Life Sciences, Bruxelles, Belgium) for 30 min at 37°C/5% CO_2_ to stain lysosomal and autophagic vesicles, respectively. Cells were washed twice with PBS and fixed with Pierce™ 1% w/v formaldehyde (Thermo Fisher Scientific) for 1h at RT, washed twice with PBS and then stored at 4°C. Prior to imaging, plates were incubated with 2 µg/ml Hoechst 33342 for 5 min at RT and imaged using a SP8WLL confocal microscope (Leica) using a 63X oil immersion objective. Each treatment condition was performed in triplicate wells and three images were taken from each well. For image analysis, lysotracker and CYTO-ID channel background was subtracted using a rolling ball algorithm with a 10 pixel radius in Fiji/ImageJ (64). Lysotracker area and CYTO-ID area were specified for each image using Cellprofiler 3.1.9 (65) and were normalized to cell count based on Hoechst 33342 staining.

### Statistical Analysis

All statistical analyses were carried out using GraphPad Prism 8 software (Graphpad Software, San Diego, CA, USA). Normal distribution of data sets was evaluated using the Shapiro-Wilk normality test. Correlation was evaluated with a Spearman’s rank correlation test. Parametric paired data were tested with a paired t-test when comparing two groups and with RM one-way ANOVA or two-way ANOVA followed by Dunnett’s multiple comparison test versus control when comparing three or more groups. Nonparametric paired data were tested with Wilcoxon matched-pairs signed rank test when comparing two groups and with Friedman’s test followed by Dunn’s multiple comparison test versus control when comparing three or more groups. Statistical tests were considered significant when p<0.05 at 95% confidence interval.

## Data Availability Statement

The original contributions presented in the study are included in the article/**Supplementary Material**. Further inquiries can be directed to the corresponding author.

## Ethics Statement

The studies involving human participants were reviewed and approved by Institutional Review Board of the Leiden University Medical Center, The Netherlands. The patients/participants provided their written informed consent to participate in this study.

## Author Contributions

CD and TO designed the project and the main conceptual ideas. CD, SS, and KW performed the experiments and analyzed the experimental data. CD and TO wrote the manuscript. TO supervised the project. All authors contributed to the article and approved the submitted version.

## Funding

This work received financial support from the Netherlands Organization for Health Research and Development (ZonMw-TOP grant 40-00812-98-14038). The funders had no role in study design, data collection and analysis, decision to publish, or preparation of the manuscript.

## Conflict of Interest

The authors declare that the research was conducted in the absence of any commercial or financial relationships that could be construed as a potential conflict of interest.

## Publisher’s Note

All claims expressed in this article are solely those of the authors and do not necessarily represent those of their affiliated organizations, or those of the publisher, the editors and the reviewers. Any product that may be evaluated in this article, or claim that may be made by its manufacturer, is not guaranteed or endorsed by the publisher.
