# Supplementary material for: Pharmacological Poly (ADP-Ribose) Polymerase Inhibitors Decrease Mycobacterium tuberculosis Survival in Human Macrophages
Source: Front Immunol. 2021 Nov 26;12:712021. doi: 10.3389/fimmu.2021.712021 (PMC8662539; doi:10.3389/fimmu.2021.712021)
Supplement: Supplementary file 1 [file DataSheet_1.pdf]

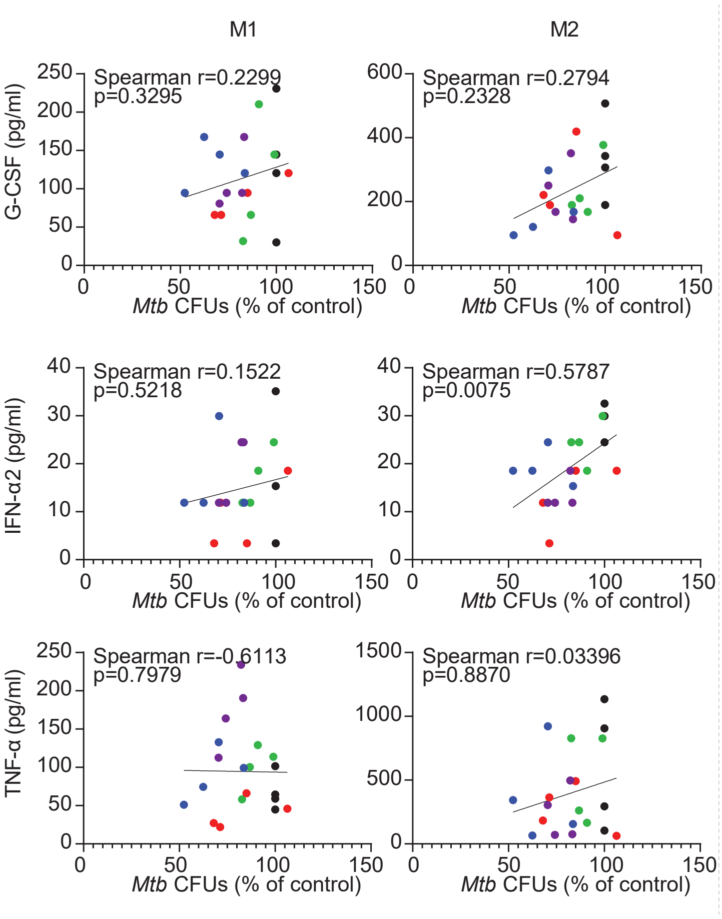

Supplemental Figure 1. Correlation plots do not identify a positive correlation between chemokine or cytokine production and *Mtb* control. Correlation between *Mtb* H37Rv outgrowth and cytokine/chemokine secretion levels in the supernatants of M1 (left) and M2 (right) cultures. *Mtb* H37Rv CFU counts are expressed as a percentage of control (i.e. DMSO). Every symbol represents data from one donor with a total of four donors. A linear regression was drawn through the data points and the correlation was determined using a Spearman's rank correlation coefficient.
