# Supplementary material for: Pharmacological Poly (ADP-Ribose) Polymerase Inhibitors Decrease Mycobacterium tuberculosis Survival in Human Macrophages
Source: Front Immunol. 2021 Nov 26;12:712021. doi: 10.3389/fimmu.2021.712021 (PMC8662539; doi:10.3389/fimmu.2021.712021)
Supplement: Supplementary file 2 [file DataSheet_2.pdf]

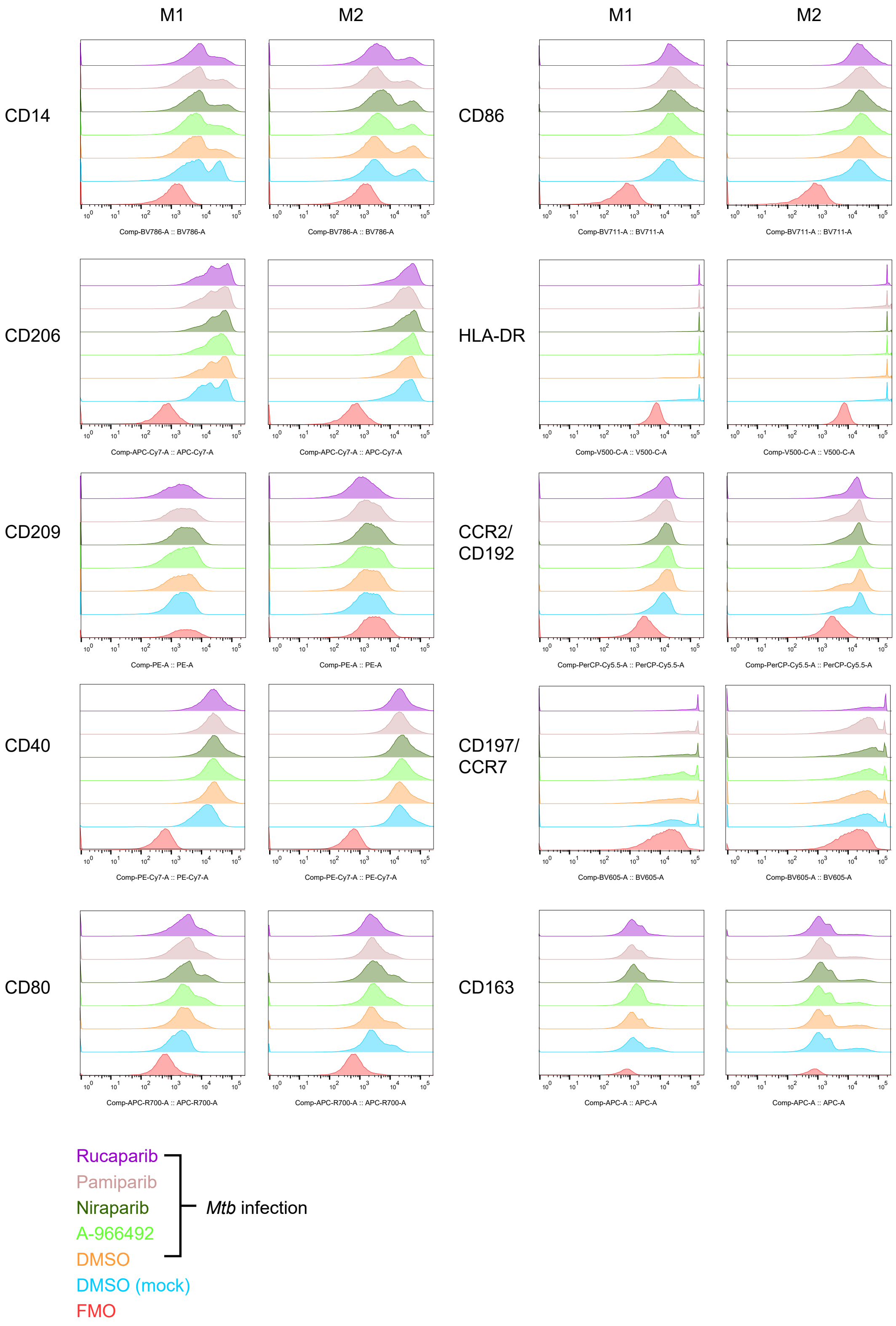

Supplemental Figure 2. Exposure to PARPi alters surface marker expression on M1 and M2 upon *Mtb* infection. Histograms showing fluorescence intensity of M1 and M2 macrophages that were either mock infected or infected with Venus-expressing *Mtb* H37Rv and treated with PARPi (10 µM) or an equal volume of vehicle control DMSO (0.1% v/v) overnight. Concatenated samples obtained from four donors are shown.
