# Supplementary material for: Pharmacological Poly (ADP-Ribose) Polymerase Inhibitors Decrease Mycobacterium tuberculosis Survival in Human Macrophages
Source: Front Immunol. 2021 Nov 26;12:712021. doi: 10.3389/fimmu.2021.712021 (PMC8662539; doi:10.3389/fimmu.2021.712021)
Supplement: Supplementary file 3 [file DataSheet_3.pdf]

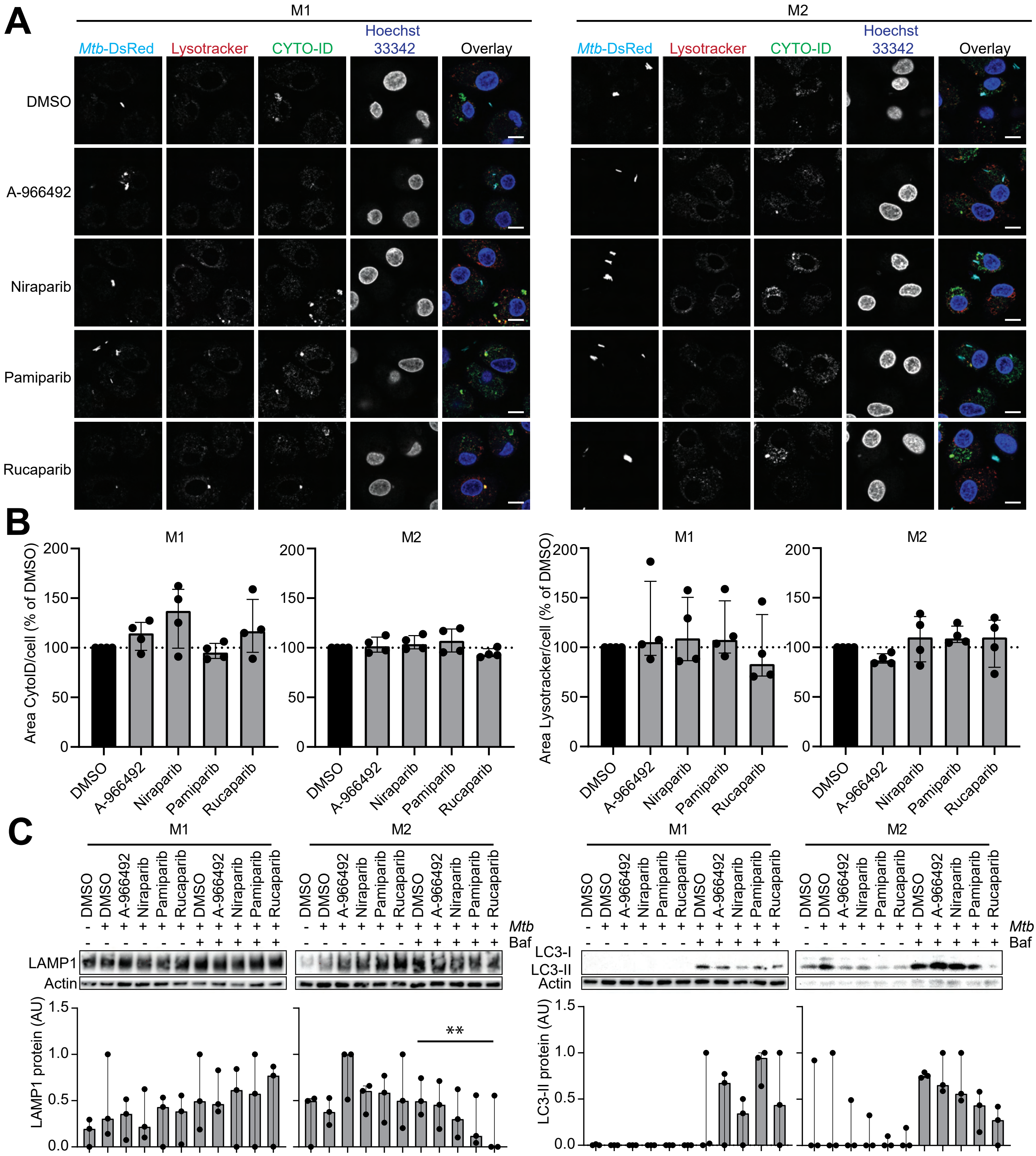

Supplemental Figure 3. A role of autophagy or lysosomal maturation in the PARPi-induced protection against Mtb could not be detected. (**A**) Confocal images of M1 and M2 infected with DsRed-expressing *Mtb* H37Rv (cyan) and stained with Lysotracker Deep Red (red), CYTO-ID green (green) and Hoechst 33342 (blue). Shown are images of one representative donor out of four donors. Scale bars annotate 10 µm. (**B**) Quantification of the CYTO-ID area per cell and the Lysotracker area per cell of DsRed-expressing *Mtb* H37Rv-infected M1 and M2 after 4h treatment with PARPi (10 µM) or an equal volume of vehicle control DMSO (0.1% v/v) . Data represent the median ± interquartile range from four different donors. Dots represent the mean from triplicate wells of a single donor. Data are expressed as percentage of control (i.e. DMSO). Differences were significant by RM one-Way ANOVA with Dunnett's multiple comparison test against DMSO. (**C**) Western immunoblot analysis of autophagic and lysosomal markers. M1 and M2 were infected with Mtb H37Rv and treated with PARPi (10 µM) or an equal volume of vehicle control DMSO (0.1% v/v) for 4h in the presence or absence of bafilomycin A1 (Baf) (10 nM). Shown are blots of one representative donor out of three donors tested and the quantified band intensity is depicted in arbitrary units (AU). Differences were significant by Friedman's test with Dunn's multiple comparison test against DMSO. ** p<0.01.
