# Supplementary material for: Pharmacological Poly (ADP-Ribose) Polymerase Inhibitors Decrease Mycobacterium tuberculosis Survival in Human Macrophages
Source: Front Immunol. 2021 Nov 26;12:712021. doi: 10.3389/fimmu.2021.712021 (PMC8662539; doi:10.3389/fimmu.2021.712021)
Supplement: Supplementary file 4 [file DataSheet_4.pdf]

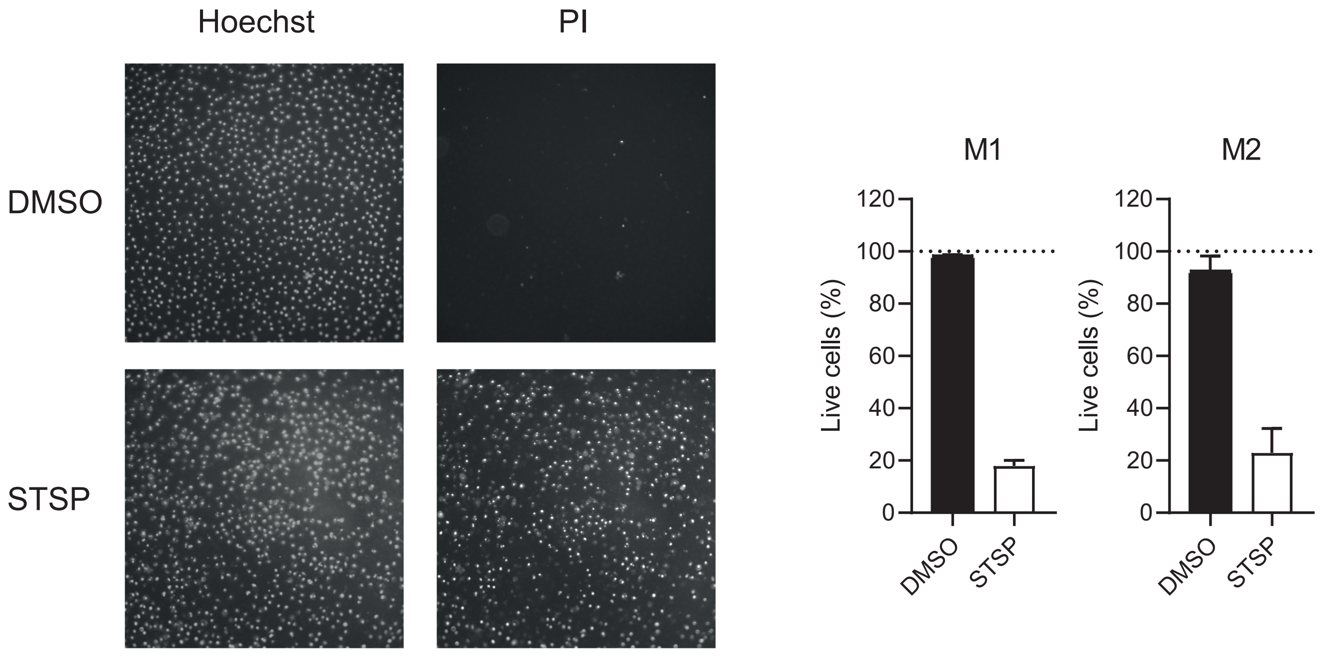

Supplemental Figure 4. Cell viability was assessed by Hoechst/PI staining. M1 and M2 were treated with DMSO or STSP (2.5 μM) overnight. Microscopy images were taken (left panel) and Hoechst-positive and PI-positive cells were quantified to determine the percentage of live cells (right panel). Shown are the means of three technical replicates from one representative donor, out of four donors tested.
